# Standards for Collection, Preservation, and Transportation of Fecal Samples in TCM Clinical Trials

**DOI:** 10.3389/fcimb.2022.783682

**Published:** 2022-04-20

**Authors:** Wenquan Su, Yawei Du, Fengmei Lian, Hui Wu, Xinrong Zhang, Wenli Yang, Yunfeng Duan, Yuanming Pan, Weijng Liu, Aiming Wu, Bowen Zhao, Chongming Wu, Shengxian Wu

**Affiliations:** ^1^ Dongzhimen Hospital, Beijing University of Chinese Medicine, Beijing, China; ^2^ Department of Endocrinology, Guang’anmen Hospital, China Academy of Chinese Medical Sciences, Beijing, China; ^3^ The First Affiliated Hospital, Guangzhou University of Chinese Medicine, Guangzhou, China; ^4^ Fangshan Hospital, Beijing University of Chinese Medicine, Beijing, China; ^5^ Institute of Microbiology, Chinese Academy of Sciences, Beijing, China; ^6^ The 7th Medical Center, Chinese People’s Liberation Army General Hospital, Beijing, China; ^7^ Beijing QuantiHealth Technology Co, Ltd, Beijing, China; ^8^ School of Chinese Materia Medica, Tianjin University of Traditional Chinese Medicine, Tianjin, China

**Keywords:** traditional Chinese medicine (TCM), clinical trial, gut microbiome, fecal sample collection, fecal sample processing, standard

## Abstract

**Background:**

Unlike chemical drugs with a single or a few kinds of active compounds, traditional Chinese medicines (TCMs)uses herbal formulas composed of numerous kinds of chemical constituents. Therefore, TCM clinical trials require unique and stricter standards for collecting, preserving, and transporting fecal samples than those used for chemical drugs. Unfortunately, there are no special standards for processing fecal samples in TCM clinical trials.

**Methods:**

We invited interdisciplinary experts within TCM clinical trials and gut microbiome research to help formulate this standard. After more than a year’s in-depth discussion and amendments, we achieved a standard *via* expert interviews, literature research, questionnaire surveys, and public opinion solicitation. This standard has been reviewed and approved by the Standards Office of China of the Association of Chinese medicine.

**Results:**

We established a sample information processing method prior to TCM clinical sample collection, which is adapted to the unique features of TCM. The method formulates detailed processing requirements for TCM information in addition to the factors that may disturb the gut microbiome. We also constructed a set of methods for collecting, preserving, and transporting fecal samples that meet the characteristics of TCM. These methods formulate detailed operating specifications on the collection approaches, storage conditions, transportation requirements, and management of fecal samples.

**Conclusions:**

This standard guides the information processing prior to sample collection and the standard operating procedures for the collection, preservation, and transportation of fecal samples in TCM clinical trials, which also can be used as a reference by clinicians and researchers in modern medicines.

## 1 Introduction

The gut microbiome is the largest and most complex micro-ecosystem of the human body (Thursby and Juge, 2017). A huge body of evidence has demonstrated that the gut microbiome strongly impacts the host’s health and is closely related to the pathological mechanisms of infections, metabolism, autoimmune disorders, cancer, and other diseases (Thaiss et al., 2016; Cani, 2018; Gopalakrishnan et al., 2018; Dabke et al., 2019). The gut microbiome and drugs interact during medical regimens (Li et al., 2009; Shin et al., 2014; Feng et al., 2015; Liu et al., 2019; Yue et al., 2019; Wu et al., 2022). The in-depth study of the interactions between the gut microbiome and medicines is an important way to reveal their mechanisms (Wang et al., 2021; Zhang et al., 2021). Due to the lack of standardized processing methods, the results from individual studies are inconsistent, and further analysis and comparison of published data are challenging. A previous clinical study has reported that BBR ameliorates diabetes by increasing the abundance of *Bifidobacterium* (Chen et al., 2016). However, more high-quality, large-scale studies have shown that BBR reduces the abundance of *Bifidobacterium* in human participants, which contradicts previous results (Sun et al., 2017).

Meta-analyses have shown that the heterogeneity of medical results on the gut microbiome is also significant (Zheng et al., 2021). Concerning the source of heterogeneity, except for interindividual variation (Falony et al., 2016) (gender, age, ethnicity, geography, and occupation), lifestyle intervention and sample processing strategies are important confounding factors. Some studies have shown that eating habits significantly impact the gut microbiome, which explains 57% of the total structural variations (Sánchez-Tapia et al., 2019). Among them, alcohol consumption and prebiotic use are particularly strong sources of gut microbiome variance (Dubinkina et al., 2017). In addition, exercise training induces compositional and functional changes in the human gut microbiome, especially in SCFA-producing taxa (*Faecalibacterium* spp. and *Lachnospira* spp.) (Allen et al., 2018). There are apparent inconsistencies between researchers and the actual fecal sample collection and preservation procedures, which is another important reason for the variability of research results (Zhang et al., 2017). Some studies have chosen to store fecal samples at -20°C, but a study suggested that feces storage at -20°C could adversely affect the abundance of *Firmicutes* and *Bacteroidetes* (Bahl et al., 2012). Collecting the outer or inner parts of the feces makes a significant difference and produces diametrically opposite results regarding the ratio of aerobes/anaerobes (Wu et al., 2019). Complex processing parameters, such as moisture content, decoction time, and temperature, influence the bioactivities of drugs, leading to discrepancies in the results. Although interindividual variation between subjects is inevitable, these technical sources of variation are controllable within clinical data set information processing and fecal sample manipulation management. At present, many studies have considered only limited confounding factors, and a systematic and comprehensive methodological standard is urgently needed.

For TCM clinical trials, TCM theories guide the formulation of diagnostic criteria and outcome indicators in TCM clinical trials (Hao et al., 2017). During information processing, it is necessary to analyze the characteristics of TCM, such as the holistic philosophy, syndrome differentiation and treatment, and drug prescriptions, in order to reveal the unique relationship between TCM and the gut microbiome. Unlike chemical drugs, TCM is often composed of compound herbal prescriptions, which have the characteristics of diverse dosage forms, large dosages, complex components, and multi-target mechanisms (Sun et al., 2013; Wang et al., 2021). The oral components of TCM with a lower bioavailability have a longer residence time within the intestine as well as more complex metabolic processes (Gong et al., 2020). The amount of TCM residual components in the feces is also significantly higher than other chemical drugs. The components that are not absorbed by the small intestine will directly contact the intestinal flora after entering the colon. These characteristics lead to a more direct effect of TCM on the fecal flora; thus, it is easier to adversely affect the structure of the fecal flora and the detection of metabolites (Chen et al., 2016). Therefore, compared with other trials, TCM clinical trials have unique and stricter requirements for information processing and operation management in obtaining fecal samples (Del Savio et al., 2017; Han et al., 2019). The authenticity of fecal samples is affected by various interference factors, including improper processing of the subject’s information, contamination of the external environment and equipment, improper storage time, temperature, and transportation methods (Ji et al., 2019). All these factors affect the accuracy of the final results; therefore, establishing standardized sample processing guidelines in order to obtain uniform and high-quality fecal samples is of great importance to TCM clinical research involving the gut microbiome. Standardization guidelines also help achieve uniform and comparable results from different experiments performed by different research groups (Deda et al., 2015; Amos et al., 2020). Currently, systematically describing the detailed processing of the fecal sample in clinical reports has been highly recommended (Neuberger-Castillo et al., 2020).

## 2 Materials and Methods

### 2.1 Study Design

**Figure 1:**
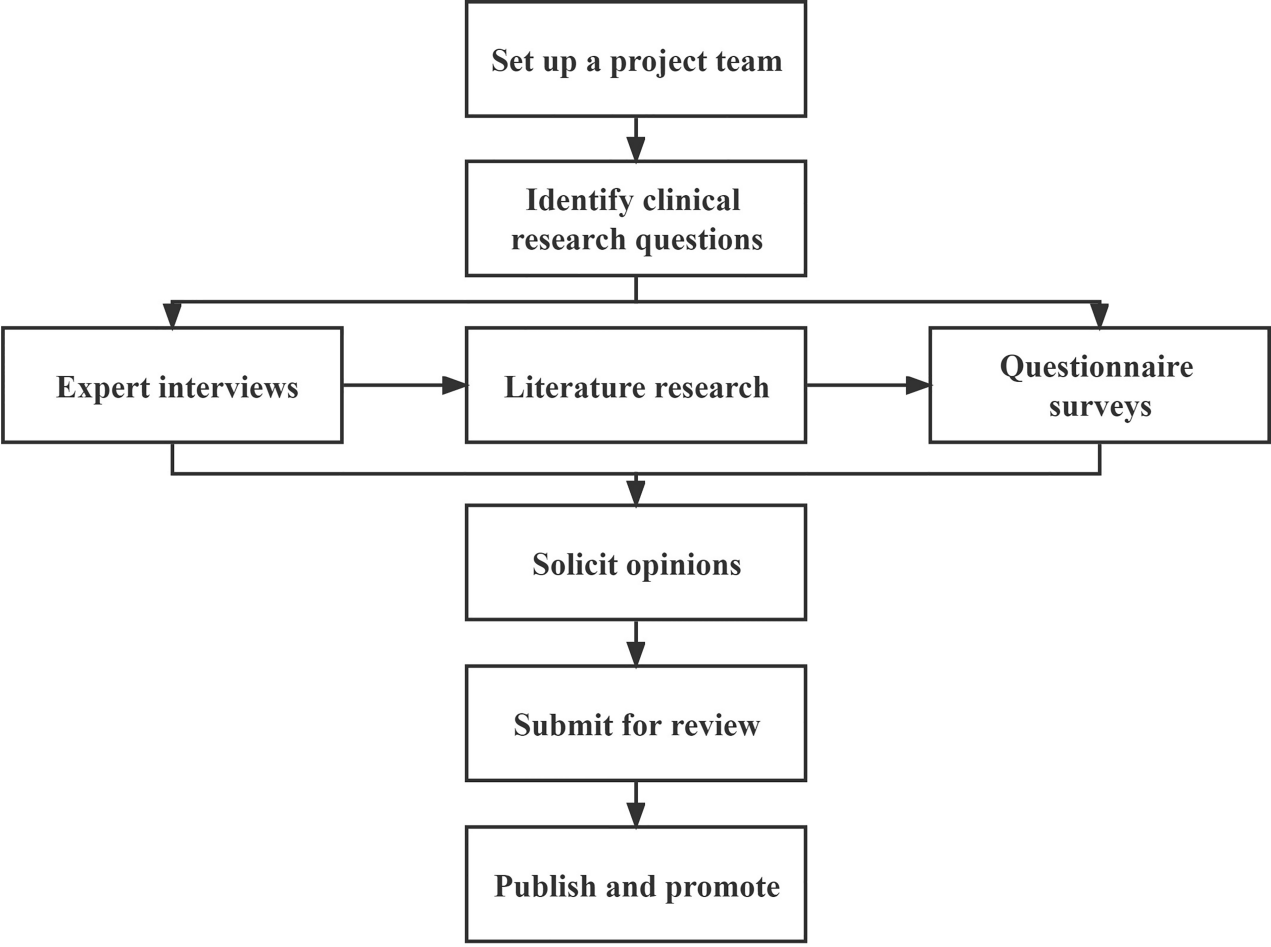
Flowchart for the development of Standards for collection, preservation, and transportation of fecal samples in TCM clinical trials.

The project team comprises 12 experts and 1 secretary within TCM clinical trials and gut microbiome research. There are a total of 8 institutions, including 5 clinical hospitals, 2 research institutes, and 1 company. The project team jointly completed the drafting of the plan for this standard and invited methodological experts to review the proposed standard. In April 2020, the project was officially established in the Standards Office of China Association of Chinese medicine. The preparation of this consensus was carried out in strict accordance with the standards steps. First, a project team was established to clarify the primary research issues, and then expert interviews, literature research, and questionnaire surveys were used to form an expert consensus. Next, this standard was further revised and improved by conducting a solicitation of opinions. Finally, submit for review after the standard revision is completed (**Figure 1**).

### 2.2 Expert Interviews

The expert interviews adopt a semi-structured interview format. A project member joint discussion determines the list of members within the interviewed expert group. A total of 4 interviewed experts, with at least 10 years of work experience, significant academic achievements within the field of TCM-microbiome research, and can provide methodological guidance on the content, format, preparation, and utility of this standard.

### 2.3 Literature Research

After the project team invited the methodological experts to discuss, the document research plan was determined, and a detailed retrieval strategy was developed. The retrieval time was from 1990 to 2020. The search tools included 3 English language databases (PubMed, SCI, and SpringerLink), 4 Chinese databases (China Knowledge Network, Weipu Science and Technology Journal Database, Wanfang Full-text Library, and Chaoxing Digital Library), a national standard information service platform, and the patent platform State Intellectual Property Office. The searched materials and information covered various forms of documentation such as journal manuscripts, master and doctoral theses, e-books, online reviews, standards, and patents. Independent personnel screened and summarized the literature as well as conducted the qualitative and quantitative analysis.

Keywords: Chinese medicine, herbal medicine, traditional medicine, clinical trials, intestinal flora, gut microbiome, fecal samples, operations, specifications.

### 2.4 Questionnaire Surveys

Based on the foundation of the previous research work, the project team used the improved Delphi method to reach a consensus through questionnaire voting. Compared with the traditional Delphi method, the improved Delphi method is based on the previous research results, replacing the open questionnaire with a structured questionnaire, which can significantly improve efficiency (Hasson et al., 2000). If the consensus degree of a proposed recommendation opinion is >75%, it is considered that the opinion has reached a consensus, and only minor modifications are required based on expert opinions. If the consensus degree of a proposed recommendation opinion is less than <75%, the opinion has not reached a consensus. A total of 14 research experts participated in the questionnaire surveys.

### 2.5 Solicited Opinions

Letters were sent out to solicit opinions, from September 21, 2020, to November 20, 2020; the draft of the soliciting opinions was sent to 30 experts from 30 institutions within 17 provinces in China. Thirty experts responded to the letter. Out of the 30, 23 experts (76.67%) had no opinion, and 7 experts replied with a suggestion or an opinion. The project team discussed and revised the suggestions and opinions individually. The revised standard was submitted for review in November 2020. The Standards Office of the Chinese Society of Chinese Medicine independently invited 10 experts to conduct a peer review. In March 2021, the results were finally approved by a unanimous vote and officially announced.

## 3 Results

### 3.1 Information Processing Before Fecal Samples Collection

#### 3.1.1 Basic Information of the Subjects

The TCM clinical trials information should be carefully reviewed and recorded, including project number, project name, research institutions, and subject identification number. Other required information includes (1) each subject’s personal information including name, gender, birthday, ethnicity, height, weight, BMI, occupation, and residence, (2) medical information including past medical history, surgical history, marriage and childbirth history, and family genetic history, and (3) infectious disease information including a history of infectious diseases and a history of contact with epidemic areas.

#### 3.1.2 Information on the Use of TCM

The production and use of TCM information should be carefully reviewed and recorded (Lin et al., 2021), including (1) the manufacturer, production batch, production date, preservation method, and processing method of the trial drug, (2) the diseases and symptoms treated with TCM, TCM name, dosage form, dosage administration method, time, and frequency of TCM administration, and (3) a history of allergies and adverse reactions to TCM.

#### 3.1.3 Information on the Use of Antibiotics

The usage information of antibiotics should be carefully reviewed and recorded (Hao et al., 2020), including the reason for use, the type of antibiotic, the dosage form, the method of administration, the intensity and frequency, and the start and last time of medication administration.

#### 3.1.4 Gastrointestinal Information

The subject’s gastrointestinal discomfort should be carefully reviewed and recorded (Weersma et al., 2020), including nausea, vomiting, bloating, acid reflux, abdominal pain, diarrhea, constipation, blood in the stool, identify the cause, frequency and intensity of uncomfortable symptoms, physical and chemical examinations, diagnosis, treatment, and the start and end time of therapy.

#### 3.1.5 Information on Other Influencing Factors

For combined medication (Glassner et al., 2020), especially medication pertaining to the gastrointestinal tract, it should be clarified whether to use microecological preparations, as well as the type, composition, dosage, time, and frequency of the drug used. For eating habits, meat-eaters and vegetarians, the use of probiotic/prebiotic food supplements and their ingredients, and the duration and frequency of smoking and drinking should be clearly distinguished (Bajaj, 2019). Exercise habits, it is necessary to distinguish between athletes and non-athletes and record the intensity, duration, and frequency of exercise (Allen et al., 2018). Mental health should be clearly distinguished among the patients with mental illness and those without and the manifestations and severity of depression and stress should be recorded.

#### 3.1.6 Fecal Condition Information

Fecal sample condition should be recorded and analyzed after the subjects defecate and before the actual sample collection, including the time, period, and frequency of defecation habits, whether assisted defecation is needed and specific usage methods, reactions and accompanying symptoms during defecation, the shape, smell, color, and residue of the feces. However, it can be combined with the TCM theories of deficiency and actuality, cold and heat, qi and blood, body fluid, and viscera location in order to conduct a TCM syndrome differentiation analysis of the feces (Zhang et al., 2019).

### 3.2 Collection of Fecal Samples

#### 3.2.1 Collection Time

Oral administration is the most common route of administration in TCM clinical trials. Fecal samples should be collected before administering the first dose of medication; it is recommended to collect fecal samples in the morning (Wu and Tan, 2019). It is best to form a fixed bowel habit to facilitate the following consistent simultaneous collection. If a fecal sample is not collected at the prescribed time, as a remedial plan, it should be collected within 2 hours after administration of the medication. In the event of acute diarrhea, constipation, or other unique circumstances, the temporary remedial collection of fecal samples should be carried out 8 hours after administration of the medicine and before the next medication administration.

For non-oral administration routes, such as intravenous and intramuscular injection, acupuncture, and massage, since they do not directly pass through the gastrointestinal tract, fecal samples can be taken at any time during the treatment. However, for direct intestinal administration such as the anus or an enema, fecal samples should be collected before the administration of therapy. It should be emphasized that the fecal sample cannot be collected immediately or prematurely after direct administration. In special cases, remedial collection can be performed 12 hours after administration. In addition, if the clinical trials have a unique research purpose, the collection time of the fecal samples can be adjusted under the principle of minimizing the TCM residue within the feces (Angelakis et al., 2016).

The frequency of fecal sampling should be collected before treatment and at least once during each treatment cycle (Miyoshi et al., 2020). Each collection should be completed within 2 days before the end of the treatment cycle.

#### 3.2.2 Collection Location

The collection of fecal samples can be done at the hospital under the guidance of the principal investigator, or it can be done at home by the subject following the collection instructions. It is recommended that fecal sampling should be completed at the hospital.

#### 3.2.3 Collection Method

Before the first collection of the fecal samples, the subjects should be fully informed of the specific operating methods and primary collection points (**Table 1**) (Bolte et al., 2021). It is recommended to use a special fecal sample kit for collection (Choo et al., 2015; Szopinska et al., 2018). If economic conditions permit, it is recommended to repeat sampling multiple times in order to reduce the relative error caused by the methodology (Fan and Pedersen, 2021).

**Table 1:** Primary points of the collection method of fecal samples in TCM clinical trials.

1	Fecal samples should be collected immediately after defecation.
2	Before sampling, the urine should be discharged to avoid contamination.
3	The feces should be collected in a dry and clean special bedpan. Avoid using sitting or squatting toilets.
4	A sterile sampling device should be used to collect the feces. There should be no disinfectant or sewage in the excrement collector. Avoid the use of metal containers.
5	The excreted feces should be classified according to the Bristol fecal classification method. It is recommended to collect 3-4 grade feces. Under special circumstances, hard feces of grades 1-2 can be collected. However, collection of sparse grade 5-7 feces should be avoided, especially grade 7 feces should not be collected unless there is a particular research need.
6	The excreted feces on the inner side of the middle and posterior segments should be taken. In order to prevent the adverse effects of feces contacting external microorganisms, the surface layer of the feces should not be taken.
7	The sample collection volume is approximately 1-2 grams. If the sample volume required for the research is larger, it should be divided into multiple collections not to exceed 1-2 grams per collection.

#### 3.2.4 Sample Management After Collection

After completing the collection, the following information should be recorded: (1) the sample’s basic information, including sample number, type, volume, and properties, (2) the entire collection process, including the informed consent, clinical questionnaire, collection location, collection method, collection time, and the participants information.

The collection of fecal samples with potentially infectious diseases should be implemented in accordance with national policies and regulations. It is necessary to adopt safe operation procedures and containers for fecal collection of the corresponding biological safety level needs.

### 3.3 Preservation of Fecal Samples

#### 3.3.1 Preservation Method

Fecal samples should be preserved using liquid nitrogen rapid freezing (Moossavi et al., 2019). For example, the fecal samples should immediately be put into liquid nitrogen for rapid freezing after being collected, and then immediately transferred to a -80°C freezer for long-term storage (Shaw et al., 2016). When quick freezing is not immediately possible, the samples should be frozen in a -80°C freezer within half an hour after the sample is collected (Gorzelak et al., 2015).

For fecal samples collected from clinical patients or remote areas, due to the lack of rapid low-temperature freezing conditions, stable liquid solutions should be used for storage, such as 100% ethanol, EDTA buffer, or commercially available reagents (Nsubuga et al., 2004; Han et al., 2018; Jenkins et al., 2018). Fecal samples using stable liquid solutions should be delivered to the clinical investigators within 2 days for uniform cryopreservation. It is recommended to use commercially available reagents for preservation.

When commercial reagents are used in sample preservation within TCM clinical trials, products produced by the same company should be used for all samples during the entire trial. Furthermore, the sample container should be filled with aliquots and a sufficient volume of reagents that cover the entire sample to ensure the consistency of sample processing. In addition, it should be noted that products from different companies cannot be mixed. The commercial reagents should be shaken and inspected before use and after adding the samples.

#### 3.3.2 Sample Management During Preservation

After the fecal samples are preserved, the preservation information, sample number, preservation method, preservation temperature, and storage location should be recorded. Using a specific and uniform format code to process, classify, and preserve the samples through a biological sample library data-based management system is recommended.

The fecal samples should not be frozen and thawed repeatedly during the cryopreservation period (Li et al., 2016). Simultaneously, the fecal samples should be regularly sampled and inspected in strict accordance with the standard operating procedures to ensure the quality of sample preservation. The fecal samples information should also be updated promptly.

The frozen preservation time of the fecal samples should be as short as possible, no more than 6 months (Carroll et al., 2012). If the storage conditions are stable, the sample can be extended to 1 year (Shaw et al., 2016). Conversely, the samples will be sent to the laboratory for testing after the first freeze-thaw cycle if the storage conditions are unstable.

Fecal samples with potentially infectious diseases should be preserved in accordance with the operating specifications within the national laws and policies. The infectious disease information of the fecal samples should be clearly marked and stored in a particular location.

### 3.4 Transportation of Fecal Samples

#### 3.4.1 Transportation Method

Researchers must first identify the fecal samples that need to be transferred to the laboratory for testing and fill in the sample information table, including sample label, transfer quantity, project name, and TCM information. Next, the transported fecal samples are packaged in three-layers. In addition, potentially infectious fecal samples should be packaged and labeled in accordance with the highest-level requirements of the WHOs *Guidance on regulations for the transport of infectious substances*.

Evaluating the influencing factors such as climate, season, time, distance, and temperature, an icebox with sufficient dry ice in a thick foam box can be used for short-distance transportation (Vandeputte et al., 2017). A dedicated medical sample transfer box should be used when the temperature is high, and the transfer conditions are poor (Yang et al., 2020). It is recommended that the transportation of fecal samples under dry ice storage conditions should be completed within 24 hours (Liang et al., 2020).

If the fecal samples are delivered by express delivery, a company with the qualifications necessary to transport medical biological samples should be used and should have cold chain transportation and safety monitoring capabilities.

#### 3.4.2 Sample Management During Transportation

The transfer-out, transportation, and transfer-in parties should jointly specify the specific operation methods of the fecal sample’s transportation, including the handover time, transportation route, and destination. It is also necessary to prepare relevant documents and instructions in accordance with the laws and regulations related to the transfer of biological samples.

When the fecal sample is transferred out or received, the following information needs to be verified and includes whether the packaging is intact, whether the packaging label is clear, and whether the information indicated in the document is consistent. After verification, the transfer record sheet should be signed for confirmation. Meanwhile, the information tracking system provided by the company can be used to locate and track the entire sample transportation process.

After the transportation of the fecal samples is completed, the information of the transfer process should be recorded and include the sample number and quantity, the time and place of transportation, and each personnel within the transfer chain. Moreover, relevant documents should be kept including the sample delivery form, the sample receipt form, and the courier notes.

For the transportation of fecal samples of potentially infectious diseases, the samples shall be classified and packaged in accordance with the requirements of local laws and policies. A dedicated person is responsible for verifying this information to ensure that the packaging, containers, and labels meet the standards. Fecal samples involving genomic information or data should be processed in accordance with national requirements (Mohammed Yakubu and Chen, 2020).

## 4 Discussion

TCM has been used in China for more than 2000 years. TCM is starting to receive widespread attention and recognition globally as a therapeutic adjuvant to contemporary and alternative medicines due to TCMs unique theoretical models and powerful therapeutic effects (Xu et al., 2013). In recent years, exploring the roles of TCM and the gut microbiome has emerged as a new frontier in understanding the utility of TCM (Feng et al., 2019). More and more studies are paying attention to the gut microbiome within TCM clinical trials. Fecal matter is the preferred source of gut microbiome samples in TCM clinical trials. Fecal samples are convenient, easy to obtain, non-invasive, and the characteristics of the microbial flora in the fecal samples are consistent with what is found in the intestinal tract (Raoult and Henrissat, 2014; Bassis et al., 2017). In several clinical trials, fecal samples have been widely used to assess the gut microbiome. High-throughput sequencing of bacterial DNA and metabolites extracted from human fecal samples have been a key step in understanding the gut microbiome’s structure, diversity, and metabolic changes (Neuberger-Castillo et al., 2020). Bacterial DNA in feces has poor stability and is prone to degradation when exposed to air or high temperature. The collection procedures, preservation temperature, preservation status, and transportation conditions all impact the diversity and integrity of the fecal samples and will ultimately influence the microbiome profile results (Vandeputte et al., 2016; Wu et al., 2019). In addition to routine operations, the processing of TCM information is an important factor that needs to be considered (Zhang et al., 2017); thus, leading to the misunderstanding of the interpretation of the test results. Low concordance between TCM clinical trials that have explored the role of the gut microbiome limits the capacity to identify causal relationships between the host-related microorganisms and the pharmacology (Vujkovic-Cvijin et al., 2020). The need for a reliable fecal sample processing protocol is essential in analyzing the results of metagenomic data of the gut microbiome in order to obtain high concordance results (Human Microbiome Project Consortium, 2012). Although researchers have expressed great interest in the contribution of TCM-microbiome interactions to human health, there is still no standardized protocol within TCM clinical trials to guarantee the sample quality of the metagenomic analysis (Wang et al., 2019). Therefore, it is critical in formulating a risk-free, standardized, effective, and safe sampling method to provide researchers with methodological guidance.

Numerous host and environmental factors can affect the gut microbiome, such as age, gender, ethnicity, drinking, mood changes, as well as medication and surgical history (Vujkovic-Cvijin et al., 2020). The collection of the contents and the analysis strategy of the microbiome associated information data sets should also consider the characteristics of TCM. According to the combinatorial principle of “King, Vassal, Assistant, and Delivery servant,” TCM is combined as a formula to treat diseases. The effect of TCM on gut microbiota is also based on the principle of “multiple components against multiple targets”. In addition, the quality and efficacy of the medicine are influenced by different techniques for extraction and isolation of TCM ( Decoction, Maceration, Percolation, Reflux extraction, Soxhlet extraction) (Zhang et al., 2018; Zhang et al., 2019). It is necessary to strictly follow the instructions to ensure the consistency of TCM quality (Sinha et al., 2015). Therefore, the production information and usage of TCM should be recorded and is essential in order to interpret the results. Different fecal textures have significant differences in the structural characteristics of the gut microbiome (Lewis and Heaton, 1997). During the TCM clinical trials, the Bristol classification chart can record the consistency and texture of the feces to effectively distinguish the types of samples from severe constipation to severe diarrhea (Bellini et al., 2017). Moreover, observing the condition of the feces is also an important reference tool to identify different TCM syndromes. Combining the fecal information such as the accompanying symptoms during defecation, the shape, smell, color, and fecal residue, TCM syndrome differentiation analysis of the feces can be performed. However, it is impractical to record all microbial-related confounding factors because the comprehensiveness and feasibility of data collection are equally important (Integrative and (iHMP) Research Network Consortium, 2014). Therefore, the controllable and significant factors within TCM clinical trials should be analyzed and recorded as often as possible.

The most “representative” collection time of fecal samples within TCM clinical trials is prior to taking the medicine in the morning. At that time the efficacy of TCM has been stabilized, and the acute stimulation of the gastrointestinal tract and flora by the drug can be avoided in order to obtain samples with a higher positive rate (Wu and Tan, 2019). Since fecal samples collected at different timepoints are an important source of heterogeneity, fixed bowel habits are conducive to forming a consistent collection timepoint and reducing the risk of bias (Choo et al., 2015). In addition, non-drug therapies such as acupuncture and massage are also commonly used methods of Chinese medicine used to treat disease (Millstine et al., 2017). After therapeutic intervention, the collection time of the fecal samples is less rigid; thus, improving the convenience of fecal sampling. Many of TCM clinical trials rely on participants collecting fecal samples at home. Some studies have indicated that understanding the purpose of TCM treatment and providing a collecting method leaflet will increase the participant willingness and the accuracy of self-collect fecal samples (Lecky et al., 2014). Therefore, we recommend that researchers should explain the procedures and reasons for these specific collection methods to participants. TCM clinical trials usually include multiple treatment cycles. Experts suggest that longitudinal surveys involving time sequence analyses of the gut microbiome relative to clinical metadata results should be used as a strategy to reduce the risk of bias in the analysis results (Miyoshi et al., 2020; Fan and Pedersen, 2021). Therefore, we recommend that the frequency of sample collection be done prior to treatment and once during each treatment cycle. Some research has found differences in the microbial structure of different fecal collection sites (Swidsinski et al., 2008). It is necessary to consider the potential variability and risks of the sampling sites (Wu et al., 2019). Thus, we emphasize that the specific collection site should be the part of the fecal sample that is not exposed to air (Hsieh et al., 2016). Numerous compounds in TCM have low bioavailability and high levels of fecal residue. Studies have shown that DNA extraction and sequencing of fecal samples requires that each sample weigh no less than 200 mg after removing the residues (Claassen et al., 2013). Therefore, in order to avoid the adverse influences of residual TCM in the feces, we recommend that the sample collection amount be approximately 1-2 grams to avoid insufficient sample size leading to errors in DNA yield and purity.

The preservation conditions of the fecal samples can significantly affect the integrity of the extracted DNA and RNA as well as the composition of the microbial community (Wu et al., 2021). A reasonable protocol on the preservation conditions is of great significance to ensure the quality of the samples. The transformation of TCM compounds depends on specific enzymes in the gut microbiome. Freezing immediately after sampling can reduce DNA degradation and enzyme activity in order to prevent the growth of residual bacteria and potential contaminants (Bahl et al., 2012). Currently, no standards stipulate the specific length of time that a stool specimen is exposed to ambient air after being discharged from the body until it freezes (Guo et al., 2016). It is usually recommended to freeze within 15 minutes after a bowel movement (Carroll et al., 2012). Meanwhile, studies have shown that fecal samples left at ambient temperature for more than 2 hours facilitates the growth of aerobic bacteria and facultative anaerobes; thus, shifting the abundance of the sample’s flora significantly (Santiago et al., 2014). Therefore, the time should be shortened as much as possible to prevent deviations within the analysis results due to bacterial inhibition or overgrowth caused by local overheating (Guo et al., 2016). Ideally, fecal samples that are frozen immediately should be shipped to the sample library as soon as possible. A temperature of -80°C is considered the optimal temperature for solidifying the proteins in the specimen, promoting the inactivation of DNA hydrolases, and helping to obtain high-quality bacterial DNA (Shaw et al., 2016; Vogtmann et al., 2017). Fecal samples stored at -80°C should avoid freeze-thaw cycles, because the ice crystals formed during the freeze-thaw process can cause cell rupture leading to DNA damage and cell apoptosis (Li et al., 2016). Numerous freeze-thaw cycles lead to poor metagenomic DNA quality and reduced bacterial diversity, which adversely affects the results of subsequent sequencing analysis. In addition to using ultra-low temperature equipment, split charging of fecal samples is an important method in order to prevent further unnecessary freeze-thaw cycles (Thomas et al., 2015). Fecal samples stored at -80°C usually do not exceed 6 months (Carroll et al., 2012). However, some studies have shown that fecal samples can maintain a stable microbial community for up to 2 years after being frozen at -80°C (Shaw et al., 2016).

In the process of fecal sample transportation from the sample library to the laboratory for testing, the transfer-out, transportation, and transfer-in parties are responsible for the samples management. Low-temperature transportation is a common practice (Yang et al., 2020). Usually, different methods such as dry ice, medical transfer box, and cold chain transportation can be utilized (Vandeputte et al., 2017). Studies have shown that 4°C refrigerated transportation can be selected when the transportation time is within 24 hours; however, some microorganisms that are susceptible to temperature will decrease or even die over time (Wu et al., 2010). So far, the current temperature range and allowable time during the transportation of fecal samples are still vague and more data is still needed to establish a consensus. Moreover, decreasing the transportation temperature and times as much as possible is beneficial in maintaining sample integrity (Human Microbiome Project Consortium, 2012). Researchers must also prevent excessive vibration, device damage, specimen contamination, and label loss during sample transfer. The development and application of information technology, information-based data management systems, and databases provide strong support for the quality control of TCM clinical trials. When economic and equipment conditions permit, data management systems should be used to record and store clinical data and sample information.

Obtaining fecal samples in TCM clinical trials involves numerous procedures, ranging from clinical information processing to sample collection, preservation, and transportation. Operational errors in each of these steps will cause strong biases in the downstream analysis results. Therefore, establishing standardized fecal sample protocols is an important measure needed to reduce the risk of bias caused by improper operations and handling (Cardona et al., 2012). At present, fecal sampling that meets the characteristics of TCM has always lacked a consensus protocol, which is a key issue that needs to be urgently solved. In order to meet the needs of TCM-microbiome research, some methodological studies have provided the basis for this consensus (Gerasimidis et al., 2016; Song et al., 2016). Therefore, we combine the characteristics of TCM clinical trials to propose strategies for dealing with the common confounding factors within clinical metadata; therefore, generating principles for the handling of fecal samples. This standardized protocol for fecal samples provides methodological guidance for studying the gut microbiome in TCM clinical trials. Meanwhile, it also provides a basis for conducting inter-research comparisons or meta-analyses, as well as integrating TCM-microbiome databases (Costea et al., 2017). Numerous problems have been resolved with the accumulation of knowledge and evidence, helping the quality control of microbiome fecal sampling research. However, the current methods still have limitations, and do not meet the demands for better quality control of the metagenomic analysis in TCM clinical trials. In the future, as more high-level evidence determines the optimal operating procedures, these guidelines will help with the revision of this research standard. It should be noted that this standard is mainly used for analysis of fecal samples collected from TCM clinical trials, including metagenomics, 16S rRNA gene amplicon sequencing, metatranscriptomics, and metabolomics. For other research purposes, such as multi-omics analysis (Whon et al., 2021), microbial culture (Lagier et al., 2018), fecal microbiota transplantation (Allegretti et al., 2019), etc., the procedure may need to be adjusted based on this standard.

## Conclusions

In this study, we have standardized a protocol in order to provide methodological guidance for the manipulation and processing of fecal samples for the study of the gut microbiome in TCM and modern medicine clinical trials and can be used as a reference for clinicians and researchers. This consensus standard has been established, reviewed, and approved by the Standards Office of China Association of Chinese medicine. We hope that the methods provided here will help investigators in TCM-microbiome research advance their studies.

## Data Availability Statement

The original contributions presented in the study are included in the article/supplementary material. Further inquiries can be directed to the corresponding authors.

## Author Contributions

WS and YaD conducted the study and drafted the manuscript. The results were processed by all the authors. SW and CW conceived and designed the study, reviewed, and edited the manuscript. All authors contributed to the article and approved the submitted version.

## Funding

This work was supported by National Major Scientific and Technological Special Project for “Significant New Drugs Development”, China (No. 2017ZX09304019).

## Conflict of Interest

Author BZ was employed by Beijing QuantiHealth Technology Co, Ltd.

The remaining authors declare that the research was conducted in the absence of any commercial or financial relationships that could be construed as a potential conflict of interest.

## Publisher’s Note

All claims expressed in this article are solely those of the authors and do not necessarily represent those of their affiliated organizations, or those of the publisher, the editors and the reviewers. Any product that may be evaluated in this article, or claim that may be made by its manufacturer, is not guaranteed or endorsed by the publisher.

## References

[B1] Allegretti J. R. Mullish B. H. Kelly C. Fischer M. (2019). The Evolution of the Use of Faecal Microbiota Transplantation and Emerging Therapeutic Indications. Lancet (Lond Engl) 394 (10196), 420–431. doi: 10.1016/S0140-6736(19)31266-8 31379333

[B2] Allen J. M. Mailing L. J. Niemiro G. M. Moore R. Cook M. D. White B. A.. (2018). Exercise Alters Gut Microbiota Composition and Function in Lean and Obese Humans. Med. Sci. Sports Exercise 50 (4), 747–757. doi: 10.1249/MSS.0000000000001495 29166320

[B3] Amos G. Logan A. Anwar S. Fritzsche M. Mate R. Bleazard T.. (2020). Developing Standards for the Microbiome Field. Microbiome 8 (1), 98. doi: 10.1186/s40168-020-00856-3 32591016 PMC7320585

[B4] Angelakis E. Bachar D. Henrissat B. Armougom F. Audoly G. Lagier J. C.. (2016). Glycans Affect DNA Extraction and Induce Substantial Differences in Gut Metagenomic Studies. Sci. Rep. 6, 26276. doi: 10.1038/srep26276 27188959 PMC4870698

[B5] Bahl M. I. Bergström A. Licht T. R. (2012). Freezing Fecal Samples Prior to DNA Extraction Affects the Firmicutes to Bacteroidetes Ratio Determined by Downstream Quantitative PCR Analysis. FEMS Microbiol. Lett. 329 (2), 193–197. doi: 10.1111/j.1574-6968.2012.02523.x 22325006

[B6] Bajaj J. S. (2019). Alcohol, Liver Disease and the Gut Microbiota. Nat. Rev. Gastroenterol. Hepatol. 16 (4), 235–246. doi: 10.1038/s41575-018-0099-1 30643227

[B7] Bassis C. M. Moore N. M. Lolans K. Seekatz A. M. Weinstein R. A. Young V. B.. (2017). Comparison of Stool Versus Rectal Swab Samples and Storage Conditions on Bacterial Community Profiles. BMC Microbiol. 17 (1), 78. doi: 10.1186/s12866-017-0983-9 28359329 PMC5374586

[B8] Bellini M. Gambaccini D. Bazzichi L. Bassotti G. Mumolo M. G. Fani B.. (2017). Bioelectrical Impedance Vector Analysis in Patients With Irritable Bowel Syndrome on a Low FODMAP Diet: A Pilot Study. Tech. Coloproctol. 21 (6), 451–459. doi: 10.1007/s10151-017-1639-3 28567692

[B9] Bolte L. A. Klaassen M. Collij V. Vich Vila A. Fu J. van der Meulen T. A.. (2021). Patient Attitudes Towards Faecal Sampling for Gut Microbiome Studies and Clinical Care Reveal Positive Engagement and Room for Improvement. PloS One 16 (4), e0249405. doi: 10.1371/journal.pone.0249405 33831035 PMC8031379

[B10] Cani P. D. (2018). Human Gut Microbiome: Hopes, Threats and Promises. Gut 67 (9), 1716–1725. doi: 10.1136/gutjnl-2018-316723 29934437 PMC6109275

[B11] Cardona S. Eck A. Cassellas M. Gallart M. Alastrue C. Dore J.. (2012). Storage Conditions of Intestinal Microbiota Matter in Metagenomic Analysis. BMC Microbiol. 12, 158. doi: 10.1186/1471-2180-12-158 22846661 PMC3489833

[B12] Carroll I. M. Ringel-Kulka T. Siddle J. P. Klaenhammer T. R. Ringel Y. (2012). Characterization of the Fecal Microbiota Using High-Throughput Sequencing Reveals a Stable Microbial Community During Storage. PloS One 7 (10), e46953. doi: 10.1371/journal.pone.0046953 23071673 PMC3465312

[B13] Chen L. Lu W. Li Y. (2016). Berberine Ameliorates Type 2 Diabetes *via* Modulation of Bifidobacterium Species, Tumor Necrosis Factor-α, and Lipopolysaccharide. Int. J. Clin. Exp. Med. 9 (6), 9365–9372.

[B14] Chen F. Wen Q. Jiang J. Li H. L. Tan Y. F. Li Y. H.. (2016). Could the Gut Microbiota Reconcile the Oral Bioavailability Conundrum of Traditional Herbs? J. Ethnopharmacol. 179, 253–264. doi: 10.1016/j.jep.2015.12.031 26723469

[B15] Choo J. M. Leong L. E. Rogers G. B. (2015). Sample Storage Conditions Significantly Influence Faecal Microbiome Profiles. Sci. Rep. 5, 16350. doi: 10.1038/srep16350 26572876 PMC4648095

[B16] Claassen S. du Toit E. Kaba M. Moodley C. Zar H. J. Nicol M. P. (2013). A Comparison of the Efficiency of Five Different Commercial DNA Extraction Kits for Extraction of DNA From Faecal Samples. J. Microbiol. Methods 94 (2), 103–110. doi: 10.1016/j.mimet.2013.05.008 23684993 PMC5809576

[B17] Costea P. I. Zeller G. Sunagawa S. Pelletier E. Alberti A. Levenez F.. (2017). Towards Standards for Human Fecal Sample Processing in Metagenomic Studies. Nat. Biotechnol. 35 (11), 1069–1076. doi: 10.1038/nbt.3960 28967887

[B18] Dabke K. Hendrick G. Devkota S. (2019). The Gut Microbiome and Metabolic Syndrome. J. Clin. Invest. 129 (10), 4050–4057. doi: 10.1172/JCI129194 31573550 PMC6763239

[B19] Deda O. Gika H. G. Wilson I. D. Theodoridis G. A. (2015). An Overview of Fecal Sample Preparation for Global Metabolic Profiling. J. Pharm. Biomed. Anal. 113, 137–150. doi: 10.1016/j.jpba.2015.02.006 25812436

[B20] Del Savio L. Prainsack B. Buyx A. (2017). Motivations of Participants in the Citizen Science of Microbiomics: Data From the British Gut Project. Genet. Med. Off. J. Am. Coll. Med. Genet. 19 (8), 959–961. doi: 10.1038/gim.2016.208 28125088

[B21] Dubinkina V. B. Tyakht A. V. Odintsova V. Y. Yarygin K. S. Kovarsky B. A. Pavlenko A. V.. (2017). Links of Gut Microbiota Composition With Alcohol Dependence Syndrome and Alcoholic Liver Disease. Microbiome 5 (1), 141. doi: 10.1186/s40168-017-0359-2 29041989 PMC5645934

[B22] Falony G. Joossens M. Vieira-Silva S. Wang J. Darzi Y. Faust K.. (2016). Population-Level Analysis of Gut Microbiome Variation. Sci. (N. Y. N. Y.) 352 (6285), 560–564. doi: 10.1126/science.aad3503 27126039

[B23] Fan Y. Pedersen O. (2021). Gut Microbiota in Human Metabolic Health and Disease. Nat. Rev. Microbiol. 19 (1), 55–71. doi: 10.1038/s41579-020-0433-9 32887946

[B24] Feng W. Ao H. Peng C. Yan D. (2019). Gut Microbiota, a New Frontier to Understand Traditional Chinese Medicines. Pharmacol. Res. 142, 176–191. doi: 10.1016/j.phrs.2019.02.024 30818043

[B25] Feng R. Shou J. W. Zhao Z. X. He C. Y. Ma C. Huang M.. (2015). Transforming Berberine Into its Intestine-Absorbable Form by the Gut Microbiota. Sci. Rep. 5, 12155. doi: 10.1038/srep12155 26174047 PMC4502414

[B26] Gerasimidis K. Bertz M. Quince C. Brunner K. Bruce A. Combet E.. (2016). The Effect of DNA Extraction Methodology on Gut Microbiota Research Applications. BMC Res. Notes 9, 365. doi: 10.1186/s13104-016-2171-7 27456340 PMC4960752

[B27] Glassner K. L. Abraham B. P. Quigley E. (2020). The Microbiome and Inflammatory Bowel Disease. J. Allergy Clin. Immunol. 145 (1), 16–27. doi: 10.1016/j.jaci.2019.11.003 31910984

[B28] Gong X. Li X. Bo A. Shi R. Y. Li Q. Y. Lei L. J.. (2020). The Interactions Between Gut Microbiota and Bioactive Ingredients of Traditional Chinese Medicines: A Review. Pharmacol. Res. 157, 104824. doi: 10.1016/j.phrs.2020.104824 32344049

[B29] Gopalakrishnan V. Helmink B. A. Spencer C. N. Reuben A. Wargo J. A. (2018). The Influence of the Gut Microbiome on Cancer, Immunity, and Cancer Immunotherapy. Cancer Cell 33 (4), 570–580. doi: 10.1016/j.ccell.2018.03.015 29634945 PMC6529202

[B30] Gorzelak M. A. Gill S. K. Tasnim N. Ahmadi-Vand Z. Jay M. Gibson D. L. (2015). Methods for Improving Human Gut Microbiome Data by Reducing Variability Through Sample Processing and Storage of Stool. PloS One 10 (8), e0134802. doi: 10.1371/journal.pone.0134802 26252519 PMC4529225

[B31] Guo Y. Li S. H. Kuang Y. S. He J. R. Lu J. H. Luo B. J.. (2016). Effect of Short-Term Room Temperature Storage on the Microbial Community in Infant Fecal Samples. Sci. Rep. 6, 26648. doi: 10.1038/srep26648 27226242 PMC4880902

[B32] Han M. Hao L. Lin Y. Li F. Wang J. Yang H.. (2018). A Novel Affordable Reagent for Room Temperature Storage and Transport of Fecal Samples for Metagenomic Analyses. Microbiome 6 (1), 43. doi: 10.1186/s40168-018-0429-0 29482661 PMC5828344

[B33] Han X. Y. Li X. Liang N. Yan Y. Q. Wang Y. Fei Y. T.. (2019). Factors Influencing the Quality of Clinical Trials on Traditional Chinese Medicine-Qualitative Interviews With Trial Auditors, Clinicians and Academic Researchers. Complementary Ther. Clin. Pract. 37, 109–114. doi: 10.1016/j.ctcp.2019.09.004 31622811

[B34] Hao P. Jiang F. Cheng J. Ma L. Zhang Y. Zhao Y. (2017). Traditional Chinese Medicine for Cardiovascular Disease: Evidence and Potential Mechanisms. J. Am. Coll. Cardiol. 69 (24), 2952–2966. doi: 10.1016/j.jacc.2017.04.041 28619197

[B35] Hao W. Z. Li X. J. Zhang P. W. Chen J. X. (2020). A Review of Antibiotics, Depression, and the Gut Microbiome. Psychiatry Res. 284, 112691. doi: 10.1016/j.psychres.2019.112691 31791704

[B36] Hasson F. Keeney S. McKenna H. (2000). Research Guidelines for the Delphi Survey Technique. J. Adv. Nurs. 32 (4), 1008–1015. doi: 10.1046/j.1365-2648.2000.t01-1-01567.x 11095242

[B37] Hsieh Y. H. Peterson C. M. Raggio A. Keenan M. J. Martin R. J. Ravussin E.. (2016). Impact of Different Fecal Processing Methods on Assessments of Bacterial Diversity in the Human Intestine. Front. Microbiol. 7. doi: 10.3389/fmicb.2016.01643 PMC5071325 27812352

[B38] Human Microbiome Project Consortium (2012). A Framework for Human Microbiome Research. Nature 486 (7402), 215–221. doi: 10.1038/nature11209 22699610 PMC3377744

[B39] Integrative H. M. P. (iHMP) Research Network Consortium (2014). The Integrative Human Microbiome Project: Dynamic Analysis of Microbiome-Host Omics Profiles During Periods of Human Health and Disease. Cell Host Microbe 16 (3), 276–289. doi: 10.1016/j.chom.2014.08.014 25211071 PMC5109542

[B40] Jenkins S. V. Vang K. B. Gies A. Griffin R. J. Jun S. R. Nookaew I.. (2018). Sample Storage Conditions Induce Post-Collection Biases in Microbiome Profiles. BMC Microbiol. 18 (1), 227. doi: 10.1186/s12866-018-1359-5 30591021 PMC6307155

[B41] Ji B. W. Sheth R. U. Dixit P. D. Huang Y. Kaufman A. Wang H. H.. (2019). Quantifying Spatiotemporal Variability and Noise in Absolute Microbiota Abundances Using Replicate Sampling. Nat. Methods 16 (8), 731–736. doi: 10.1038/s41592-019-0467-y 31308552 PMC7219825

[B42] Lagier J. C. Dubourg G. Million M. Cadoret F. Bilen M. Fenollar F.. (2018). Culturing the Human Microbiota and Culturomics. Nat. Rev. Microbiol. 16, 540–550. doi: 10.1038/s41579-018-0041-0 29937540

[B43] Lecky D. M. Hawking M. K. McNulty C. A. ESBL steering group (2014). Patients' Perspectives on Providing a Stool Sample to Their GP: A Qualitative Study. Br. J. Gen. Pract. J. R. Coll. Gen. Practitioners 64 (628), e684–e693. doi: 10.3399/bjgp14X682261 PMC4220220 25348992

[B44] Lewis S. J. Heaton K. W. (1997). Stool Form Scale as a Useful Guide to Intestinal Transit Time. Scandinavian J. Gastroenterol. 32 (9), 920–924. doi: 10.3109/00365529709011203 9299672

[B45] Liang Y. Dong T. Chen M. He L. Wang T. Liu X.. (2020). Systematic Analysis of Impact of Sampling Regions and Storage Methods on Fecal Gut Microbiome and Metabolome Profiles. mSphere 5 (1), e00763–e00719. doi: 10.1128/mSphere.00763-19 31915218 PMC6952195

[B46] Lin T. L. Lu C. C. Lai W. F. Wu T. S. Lu J. J. Chen Y. M.. (2021). Role of Gut Microbiota in Identification of Novel TCM-Derived Active Metabolites. Protein Cell 12 (5), 394–410. doi: 10.1007/s13238-020-00784-w 32929698 PMC8106560

[B47] Li Y. Poroyko V. Yan Z. Pan L. Feng Y. Zhao P.. (2016). Characterization of Intestinal Microbiomes of Hirschsprung's Disease Patients With or Without Enterocolitis Using Illumina-MiSeq High-Throughput Sequencing. PloS One 11 (9), e0162079. doi: 10.1371/journal.pone.0162079 27603009 PMC5014423

[B48] Liu C. S. Liang X. Wei X. H. Jin Z. Chen F. L. Tang Q. F.. (2019). Gegen Qinlian Decoction Treats Diarrhea in Piglets by Modulating Gut Microbiota and Short-Chain Fatty Acids. Front. Microbiol. 10. doi: 10.3389/fmicb.2019.00825 PMC6482297 31057525

[B49] Li H. Zhou M. Zhao A. Jia W. (2009). Traditional Chinese Medicine: Balancing the Gut Ecosystem. Phytother. Res. PTR 23 (9), 1332–1335. doi: 10.1002/ptr.2590 19253310

[B50] Millstine D. Chen C. Y. Bauer B. (2017). Complementary and Integrative Medicine in the Management of Headache. BMJ (Clin. Res. ed.) 357, j1805. doi: 10.1136/bmj.j1805 28512119

[B51] Miyoshi J. Rao M. C. Chang E. B. (2020). Navigating the Human Gut Microbiome: Pathway to Success From Lessons Learned. Gastroenterology 159 (6), 2019–2024. doi: 10.1053/j.gastro.2020.09.002 33181127 PMC8546501

[B52] Mohammed Yakubu A. Chen Y. P. (2020). Ensuring Privacy and Security of Genomic Data and Functionalities. Briefings Bioinf. 21 (2), 511–526. doi: 10.1093/bib/bbz013 30759195

[B53] Moossavi S. Engen P. A. Ghanbari R. Green S. J. Naqib A. Bishehsari F.. (2019). Assessment of the Impact of Different Fecal Storage Protocols on the Microbiota Diversity and Composition: A Pilot Study. BMC Microbiol. 19 (1), 145. doi: 10.1186/s12866-019-1519-2 31253096 PMC6599303

[B54] Neuberger-Castillo L. Hamot G. Marchese M. Sanchez I. Ammerlaan W. Betsou F. (2020). Method Validation for Extraction of DNA From Human Stool Samples for Downstream Microbiome Analysis. Biopreserv. Biobanking 18 (2), 102–116. doi: 10.1089/bio.2019.0112 31999474

[B55] Nsubuga A. M. Robbins M. M. Roeder A. D. Morin P. A. Boesch C. Vigilant L. (2004). Factors Affecting the Amount of Genomic DNA Extracted From Ape Faeces and the Identification of an Improved Sample Storage Method. Mol. Ecol. 13 (7), 2089–2094. doi: 10.1111/j.1365-294X.2004.02207.x 15189228

[B56] Raoult D. Henrissat B. (2014). Are Stool Samples Suitable for Studying the Link Between Gut Microbiota and Obesity? Eur. J. Epidemiol. 29 (5), 307–309. doi: 10.1007/s10654-014-9905-4 24838696

[B57] Sánchez-Tapia M. Tovar A. R. Torres N. (2019). Diet as Regulator of Gut Microbiota and its Role in Health and Disease. Arch. Med. Res. 50 (5), 259–268. doi: 10.1016/j.arcmed.2019.09.004 31593850

[B58] Santiago A. Panda S. Mengels G. Martinez X. Azpiroz F. Dore J.. (2014). Processing Faecal Samples: A Step Forward for Standards in Microbial Community Analysis. BMC Microbiol. 14, 112. doi: 10.1186/1471-2180-14-112 24884524 PMC4021188

[B59] Shaw A. G. Sim K. Powell E. Cornwell E. Cramer T. McClure Z. E.. (2016). Latitude in Sample Handling and Storage for Infant Faecal Microbiota Studies: The Elephant in the Room? Microbiome 4 (1), 40. doi: 10.1186/s40168-016-0186-x 27473284 PMC4967342

[B60] Shin N. R. Lee J. C. Lee H. Y. Kim M. S. Whon T. W. Lee M. S.. (2014). An Increase in the Akkermansia Spp. Population Induced by Metformin Treatment Improves Glucose Homeostasis in Diet-Induced Obese Mice. Gut 63 (5), 727–735. doi: 10.1136/gutjnl-2012-303839 23804561

[B61] Sinha R. Abnet C. C. White O. Knight R. Huttenhower C. (2015). The Microbiome Quality Control Project: Baseline Study Design and Future Directions. Genome Biol. 16, 276. doi: 10.1186/s13059-015-0841-8 26653756 PMC4674991

[B62] Song S. J. Amir A. Metcalf J. L. Amato K. R. Xu Z. Z. Humphrey G.. (2016). Preservation Methods Differ in Fecal Microbiome Stability, Affecting Suitability for Field Studies. mSystems 1 (3), e00021–e00016. doi: 10.1128/mSystems.00021-16 PMC5069758 27822526

[B63] Sun D. Z. Li S. D. Liu Y. Zhang Y. Mei R. Yang M. H. (2013). Differences in the Origin of Philosophy Between Chinese Medicine and Western Medicine: Exploration of the Holistic Advantages of Chinese Medicine. Chin. J. Integr. Med. 19 (9), 706–711. doi: 10.1007/s11655-013-1435-5 23975136 PMC7089096

[B64] Sun R. Yang N. Kong B. Cao B. Feng D. Yu X.. (2017). Orally Administered Berberine Modulates Hepatic Lipid Metabolism by Altering Microbial Bile Acid Metabolism and the Intestinal FXR Signaling Pathway. Mol. Pharmacol. 91 (2), 110–122. doi: 10.1124/mol.116.106617 27932556 PMC5267522

[B65] Swidsinski A. Loening-Baucke V. Vaneechoutte M. Doerffel Y. (2008). Active Crohn's Disease and Ulcerative Colitis can be Specifically Diagnosed and Monitored Based on the Biostructure of the Fecal Flora. Inflammatory Bowel Dis. 14 (2), 147–161. doi: 10.1002/ibd.20330 18050295

[B66] Szopinska J. W. Gresse R. van der Marel S. Boekhorst J. Lukovac S. van Swam I.. (2018). Reliability of a Participant-Friendly Fecal Collection Method for Microbiome Analyses: A Step Towards Large Sample Size Investigation. BMC Microbiol. 18 (1), 110. doi: 10.1186/s12866-018-1249-x 30189859 PMC6127955

[B67] Thaiss C. A. Zmora N. Levy M. Elinav E. (2016). The Microbiome and Innate Immunity. Nature 535 (7610), 65–74. doi: 10.1038/nature18847 27383981

[B68] Thomas V. Clark J. Doré J. (2015). Fecal Microbiota Analysis: An Overview of Sample Collection Methods and Sequencing Strategies. Future Microbiol. 10 (9), 1485–1504. doi: 10.2217/fmb.15.87 26347019

[B69] Thursby E. Juge N. (2017). Introduction to the Human Gut Microbiota. Biochem. J. 474 (11), 1823–1836. doi: 10.1042/BCJ20160510 28512250 PMC5433529

[B70] Vandeputte D. Falony G. Vieira-Silva S. Tito R. Y. Joossens M. Raes J. (2016). Stool Consistency is Strongly Associated With Gut Microbiota Richness and Composition, Enterotypes and Bacterial Growth Rates. Gut 65 (1), 57–62. doi: 10.1136/gutjnl-2015-309618 26069274 PMC4717365

[B71] Vandeputte D. Tito R. Y. Vanleeuwen R. Falony G. Raes J. (2017). Practical Considerations for Large-Scale Gut Microbiome Studies. FEMS Microbiol. Rev. 41 (Supp_1), S154–S167. doi: 10.1093/femsre/fux027 28830090 PMC7207147

[B72] Vogtmann E. Chen J. Amir A. Shi J. Abnet C. C. Nelson H.. (2017). Comparison of Collection Methods for Fecal Samples in Microbiome Studies. Am. J. Epidemiol. 185 (2), 115–123. doi: 10.1093/aje/kww177 27986704 PMC5253972

[B73] Vujkovic-Cvijin I. Sklar J. Jiang L. Natarajan L. Knight R. Belkaid Y. (2020). Host Variables Confound Gut Microbiota Studies of Human Disease. Nature 587 (7834), 448–454. doi: 10.1038/s41586-020-2881-9 33149306 PMC7677204

[B74] Wang J. S. Dai H. H. Zhang K. G. Cao K. G. Deng S. Bao B. H.. (2021). Mechanism of Huoxue Tongluo Decoction in Treatment of Erectile Dysfunction Caused by Ischemic Stroke Based on Network Pharmacology. Chin. Herbal Medicines 13 (03), 351–358. doi: 10.1016/j.chmed.2021.04.016 PMC9476638 36118932

[B75] Wang J. Feng W. Tang F. Ao H. Peng C. (2019). Gut Microbial Transformation, a Potential Improving Factor in the Therapeutic Activities of Four Groups of Natural Compounds Isolated From Herbal Medicines. Fitoterapia 138, 104293. doi: 10.1016/j.fitote.2019.104293 31398447

[B76] Weersma R. K. Zhernakova A. Fu J. (2020). Interaction Between Drugs and the Gut Microbiome. Gut 69 (8), 1510–1519. doi: 10.1136/gutjnl-2019-320204 32409589 PMC7398478

[B77] Whon T. W. Shin N. R. Kim J. Y. Roh S. W. (2021). Omics in Gut Microbiome Analysis. J. Microbiol. (Seoul Korea) 59 (3), 292–297. doi: 10.1007/s12275-021-1004-0 33624266

[B78] Wu W. K. Chen C. C. Panyod S. Chen R. A. Wu M. S. Sheen L. Y.. (2019). Optimization of Fecal Sample Processing for Microbiome Study - The Journey From Bathroom to Bench. J. Formosan Med. Assoc. Taiwan Yi Zhi 118 (2), 545–555. doi: 10.1016/j.jfma.2018.02.005 29490879

[B79] Wu C. Chen T. Xu W. Zhang T. Pei Y. Yang Y.. (2021). The Maintenance of Microbial Community in Human Fecal Samples by a Cost Effective Preservation Buffer. Sci. Rep. 11 (1), 13453. doi: 10.1038/s41598-021-92869-7 34188136 PMC8242035

[B80] Wu G. D. Lewis J. D. Hoffmann C. Chen Y. Y. Knight R. Bittinger K.. (2010). Sampling and Pyrosequencing Methods for Characterizing Bacterial Communities in the Human Gut Using 16S Sequence Tags. BMC Microbiol. 10, 206. doi: 10.1186/1471-2180-10-206 20673359 PMC2921404

[B81] Wu X. M. Tan R. X. (2019). Interaction Between Gut Microbiota and Ethnomedicine Constituents. Natural Prod. Rep. 36 (5), 788–809. doi: 10.1039/c8np00041g 30534698

[B82] Wu C. M. Zhao Y. Zhang Y. Y. Yang Y. N. Su W. Q. Yang Y. Y.. (2022). Gut Microbiota Specifically Mediates the Anti-Hypercholesterolemic Effect of Berberine (BBR) and Facilitates to Predict BBR’s Cholesterol-Decreasing Efficacy in Patients. J. Adv. Res. 37, 197–208. doi: 10.1016/j.jare.2021.07.011 35499044 PMC9039652

[B83] Xu Q. Bauer R. Hendry B. M. Fan T. P. Zhao Z. Duez P.. (2013). The Quest for Modernisation of Traditional Chinese Medicine. BMC Complementary Altern. Med. 13, 132. doi: 10.1186/1472-6882-13-132 PMC3689083 23763836

[B84] Yang L. Hou K. Zhang B. Ouyang C. Lin A. Xu S.. (2020). Preservation of the Fecal Samples at Ambient Temperature for Microbiota Analysis With a Cost-Effective and Reliable Stabilizer EffcGut. Sci. Total Environ. 741, 140423. doi: 10.1016/j.scitotenv.2020.140423 32615432

[B85] Yue S. J. Wang W. X. Yu J. G. Chen Y. Y. Shi X. Q. Yan D.. (2019). Gut Microbiota Modulation With Traditional Chinese Medicine: A System Biology-Driven Approach. Pharmacol. Res. 148, 104453. doi: 10.1016/j.phrs.2019.104453 31541688

[B86] Zhang Y. L. Cai L. T. Qi J. Y. Lin Y. Z. Dai Y. C. Jiao N.. (2019). Gut Microbiota Contributes to the Distinction Between Two Traditional Chinese Medicine Syndromes of Ulcerative Colitis. World J. Gastroenterol. 25 (25), 3242–3255. doi: 10.3748/wjg.v25.i25.3242 31333315 PMC6626730

[B87] Zhang S. Cao X. Huang H. (2017). Sampling Strategies for Three-Dimensional Spatial Community Structures in IBD Microbiota Research. Front. Cell. Infect. Microbiol. 7. doi: 10.3389/fcimb.2017.00051 PMC5323387 28286741

[B88] Zhang Q. W. Lin L. G. Ye W. C. (2018). Techniques for Extraction and Isolation of Natural Products: A Comprehensive Review. Chin. Med. 13, 20. doi: 10.1186/s13020-018-0177-x 29692864 PMC5905184

[B89] Zhang H. Y. Tian J. X. Lian F. M. Li M. Liu W. K. Zhen Z.. (2021). Therapeutic Mechanisms of Traditional Chinese Medicine to Improve Metabolic Diseases *via* the Gut Microbiota. Biomed. Pharmacother. Biomed. Pharmacother. 133, 110857. doi: 10.1016/j.biopha.2020.110857 33197760

[B90] Zhang R. Zhu X. Bai H. Ning K. (2019). Network Pharmacology Databases for Traditional Chinese Medicine: Review and Assessment. Front. Pharmacol. 10. doi: 10.3389/fphar.2019.00123 PMC6393382 30846939

[B91] Zheng Y. Ding Q. Wei Y. Gou X. Tian J. Li M.. (2021). Effect of Traditional Chinese Medicine on Gut Microbiota in Adults With Type 2 Diabetes: A Systematic Review and Meta-Analysis. Phytomed. Int. J. Phytother. Phytopharmacol. 88, 153455. doi: 10.1016/j.phymed.2020.153455 33478831

